# Efficacy of *Lactobacillus reuteri* DSM 17938 for infantile colic: Systematic review with network meta-analysis

**DOI:** 10.1097/MD.0000000000009375

**Published:** 2017-12-22

**Authors:** Pedro Gutiérrez-Castrellón, Flavia Indrio, Alexis Bolio-Galvis, Carlos Jiménez-Gutiérrez, Irma Jimenez-Escobar, Gabriel López-Velázquez

**Affiliations:** a Center for Translational Research on Early Programming Nutrition and Mother-Child Nutrition, Hospital General Dr Manuel Gea González & Dirección de Investigación. Universidad Tecnológica de México-Unitec México; b University of Bari, Bari, Italy; c Universidad Tecnológica de México-Unitec, México; d Center for Analysis on Health Evidence, Hospital General Dr. Manuel Gea González; e Head of Medical Division, Hospital General Dr Manuel Gea González; f Genetic Biochemistry Department, Instituto Nacional de Pediatria.

**Keywords:** infantile colic, *L reuteri* DSM 17938, network meta-analysis

## Abstract

**Background:**

5% to 40% of infants cry excessively, usually accompanied by fussiness and excessive of gas. There are no uniform criteria for treatment of infantile colic. *Lactobacillus reuteri* DSM 17938 has been used with promising results. The objective of this network-meta-analysis (NMA) is to compare the efficacy of *L reuteri* DSM 17938 with other interventions for infantile colic.

**Methods:**

RCTs, published between 1960 and 2015 for the treatment of infantile colic were included. Primary outcome was duration of crying after 21 to 28 days of treatment. Different databases were searched. Information was analyzed using control group as central axis. A random effect model was used. Hedges standard mean difference (SMD) and odds ratio (OR) were calculated. A SUCRA analysis was performed to evaluate superiority for each intervention.

**Results:**

32 RCTs were analyzed, including 2242 patients. Studies with *L reuteri* DSM 17938 versus Ctrl., Diet versus Ctrl. and Acupuncture versus Ctrl. were the most influential studies in the NMA. *L reuteri* DSM 17938 [WMD −51.3 h (CI95% −72.2 to −30.5 h), *P* .0001] and dietetic approaches [WMD −37.4 h (CI95% −56.1 to −18.7 h), *P* .0001] were superior compared to the other treatments.

**Conclusions:**

*L reuteri DSM 17938* and some dietetic approaches are better to other interventions for treatment of infantile colic.

## 1 Introduction

Crying is generally thought to be a normal behavior during infancy, serving as an infant's means of survival. Through crying, infants can alert to and elicit help for problems, such as hunger, soiled diapers, harsh temperature, and discomfort or pain.^[1,2]^ However, 5% to 40% of infants cry inconsolably and excessively, and this can be accompanied by bouts of fussiness and passing of gas.^[3–5]^ Wessel et al^[6]^ coined the term “infantile colic” to describe a fussy infant with colic as one who is otherwise healthy and well-fed, but with paroxysms of irritability, fussing or crying, lasting for a total of at least 3 hours a day, occurring on more than 3 days a week for a period of 3 weeks.^[7]^ In 2006, Rome III criteria was published modifying these criteria to consider the diagnosis of “infantile colic” applicable to infants with paroxysms of irritability, fussing, or crying that start and stop without obvious cause, lasting 3 or more hours per day and occurring at least 3 days per week, but for at least 1 week and no failure to thrive.^[8]^ Infantile colic can manifest as early as 1 to 2 weeks of age, with peak crying duration and fussiness typically between 6 and 8 weeks of age, and diminishing gradually until disappearing between 3 and 4 months of age.^[1,6,9,10]^ The exact etiology of infantile colic remains elusive; however, various theories have been proposed, some of which include overproduction of intestinal gas, forceful intestinal contraction, miscommunication between brain and intestine, hypersensitivity to cow's milk protein, transient lactase deficiency, negative or inadequate maternal–infant bonding or parental overstimulation, difficult infant temperament, insecure parental attachment, or changes in intestinal microbiota.^[2,6,7,10–12]^ Diverse studies have identified different microbiota patterns between infants with/without colic, which seems to affect intestinal fatty acid profiles.^[12–16]^ In 2004, Savino et al^[15]^ evaluated intestinal microflora in breastfed colicky and noncolicky infants. Seventy-one breastfed infants, aged 3.2 ± 0.6 weeks old, free from episodes of gastroenteritis and without previous use of antibiotics and probiotics, were enrolled in the study. They were divided into 2 groups: colicky (42 cases) and noncolicky (29 cases). Colicky infants were less frequently colonized by *Lactobacillus* spp., and more frequently by anaerobic gram-negative bacteria. Additionally, it seems that colicky babies are more frequently colonized with the gas-forming *Clostridium difficile*, *Escherichia* spp, and/or *Klebsiella* spp.^[17,18]^ From a therapeutic point of view, there are no uniform criteria for a specific therapeutic regimen for infantile colic. The first recommended step is to look for potential “red flags.” In 2013 Vandenplas et al^[19]^ published different algorithms for practical approach of gastrointestinal functional disorders. In this paper, the authors pointed out the importance of identifying signs/symptoms such as arching (Sandifer), GI bleeding or failure to thrive which could be associated to organic disease. If no red flags are apparent, it is recommendable to evaluate the feeding technique; then, reassure the caregivers and offer general advice, emphasizing the self-limiting nature of the condition. For breast-fed infants, clinicians should advise mothers to continue breast-feeding, with some authors recommending that nursing mothers should omit cow's milk protein (CMP) intake. The elimination diet should be continued for a minimum of 2 weeks and should continue if the infant responds well. For formula-fed infants, other authors have recommended the use of hydrolyzed and low protein-content infant formula.^[20]^ Considering the evidence about the microbiota pattern in these infants, diverse authors have published different randomized clinical controlled trials (RCTs) where the ability of *Lactobacillus reuteri* to reduce crying time in these infants has been evaluated.^[21–26]^ In 2010, Savino et al conducted an RCT to test the efficacy of this strain on infantile colic and to evaluate its relationship to the gut microbiota. Fifty exclusively breastfed colicky infants, diagnosed according to modified Wessel's criteria, were randomly assigned to receive either *L reuteri* DSM 17938 (10^8^ colony-forming units) or placebo daily for 21 days. Parental questionnaires monitored daily crying time and adverse effects. Forty-six infants (*L reuteri* group: 25; placebo group: 21) completed the trial. Daily crying times in minutes/day (median [interquartile range]) were 370 (120) vs 300 (150) (*P* = .127) on day 0 and 35.0 (85) vs 90.0 (148) (*P* = .022) on day 21 with no differences in weight gain, stooling frequency, or incidence of constipation or regurgitation between groups, and no adverse events related to the supplementation were observed.^[22]^ Three years later, Sajewska et al published with a similar design a second RCT in 80 infants aged < 5 months, identifying that the rate of responders to treatment was significantly higher in the probiotic group compared with the placebo group at day 7 (*P* = .026), at day 14 (relative risk [RR] 4.3, 95% CI 2.3–8.7), at day 21 (RR 2.7, 95% CI 1.85−4.1), and at day 28 (RR 2.5, 95% CI 1.8−3.75).^[23]^ After these RCTs, 3 additional RCTs were published: 2 with similar results in support of *L reuteri* DSM 17938^[25,26]^ and 1, a very controversial RCT with similar effects between *L reuteri* and placebo.^[24]^ Additionally, other therapeutic strategies have been used, including the use of dicyclomine, cimetropium or simethicone;^[27–32]^ infant formulas with the addition of hydrolyzed protein, soy protein, low-protein and/or prebiotics;^[33–39]^ some herbal products;^[40–43]^ acupuncture, chiropractic techniques, spinal massages, support to family/caregivers, counseling therapies, car rides during colic episodes, and/or decrease of stimulating actions.^[44–56]^ Considering there are some conflicting results related to the use of some of these strategies, the aim of this paper is to compare the efficacy of *L reuteri* DSM 17938 with other plausible therapeutic approaches for infantile colic, through a systematic review with network meta-analysis (NMA) approach attempting to identify on an evidence-based analysis which could be the best therapeutic choice.

## 2 Methods

### 2.1 Study protocol register and search strategy

This systematic review was assembled considering The PRISMA Extension Statement for Reporting of Systematic Reviews Incorporating Network Meta-analyses of Health Care Interventions^[57]^ and approved by the Internal Review Board (IRB) of the Hospital General Dr Manuel Gea González, México. We included in this review only double-blind, randomized, controlled clinical trials (RTCs), published between January, 1960 and August, 2015 in English or Spanish language. A systematic and exhaustive search was conducted in Medline, Embase, Cumulative Index to Nursing and Allied Health (CINAHL), PsycINFO, the Cochrane Central Register of Controlled Trials, Lilacs, Artemisa and in the databases of the principal international regulatory agencies in order to identify relevant studies published between 1960 and August 2015. PubMed searching algorithms was ((“infant” [MeSH Terms] OR “infant” [All Fields]) OR infantile [All Fields]) AND (“colic” [MeSH Terms] OR “colic” [All Fields]) AND ((“probiotics” [MeSH Terms] OR “probiotics” [All Fields]) OR ((“infant” [MeSH Terms] OR “infant” [All Fields]) AND formula [All Fields]) OR (“diet” [MeSH Terms] OR “diet” [All Fields]) OR (“pharmaceutical preparations” [MeSH Terms] OR (“pharmaceutical” [All Fields] AND “preparations” [All Fields]) OR “pharmaceutical preparations” [All Fields] OR “drugs” [All Fields]) OR (((“protons” [MeSH Terms] OR “protons” [All Fields] OR “proton” [All Fields]) AND pump [All Fields] AND (“antagonists and inhibitors” [Subheading] OR (“antagonists” [All Fields] AND “inhibitors” [All Fields]) OR “antagonists and inhibitors” [All Fields] OR “inhibitors” [All Fields])) OR (“dicyclomine” [MeSH Terms] OR “dicyclomine” [All Fields]) OR (“dicyclomine” [MeSH Terms] OR “dicyclomine” [All Fields] OR “dicycloverine” [All Fields]) OR (“cimetropium” [Supplementary Concept] OR “cimetropium” [All Fields]) OR (“simethicone” [MeSH Terms] OR “simethicone” [All Fields])) OR ((familiar [All Fields] OR (“caregivers” [MeSH Terms] OR “caregivers” [All Fields])) AND support[All Fields]) OR ((“counselling” [All Fields] OR “counseling” [MeSH Terms] OR “counseling” [All Fields]) AND (“therapeutics” [MeSH Terms] OR “therapeutics” [All Fields] OR “therapies” [All Fields])) OR car-rides [All Fields] OR (stimulating [All Fields] AND actions [All Fields]) OR (“chiropractic” [MeSH Terms] OR “chiropractic” [All Fields]) OR (“massage” [MeSH Terms] OR “massage” [All Fields] OR “massages” [All Fields]) OR (“acupuncture” [MeSH Terms] OR “acupuncture” [All Fields] OR “acupuncture therapy” [MeSH Terms] OR (“acupuncture” [All Fields] AND “therapy” [All Fields]) OR “acupuncture therapy” [All Fields]) OR herbal [All Fields]) AND (Clinical Trial[ptyp] AND (“1960/01/01” [PDAT]: “2015/08/31” [PDAT]) AND “humans” [MeSH Terms] AND (English[lang] OR Spanish[lang])).

### 2.2 Study selection and outcome measures

RCTs that compared the use of *L reuteri*; use of extensively or partially hydrolyzed formulas, isolated soy protein formulas, low-protein or lactose-free formulas or prebiotic added infant formulas; dicyclomine, cimetropium, or simethicone; familiar caregivers’ support, counseling therapies; car-rides interventions during colic episodes, decrease of stimulating actions, chiropractic techniques, spinal massages; acupuncture or use of herbal options versus placebo or active treatment in outpatient infantile colic infants less than 6 months old were selected for this network meta-analysis. All different treatments were included in 9 nodes (Table 1). Primary outcome analyzed was the duration of crying (in hours) observed 7 to 28 days after the beginning of treatment.

**Table 1:**
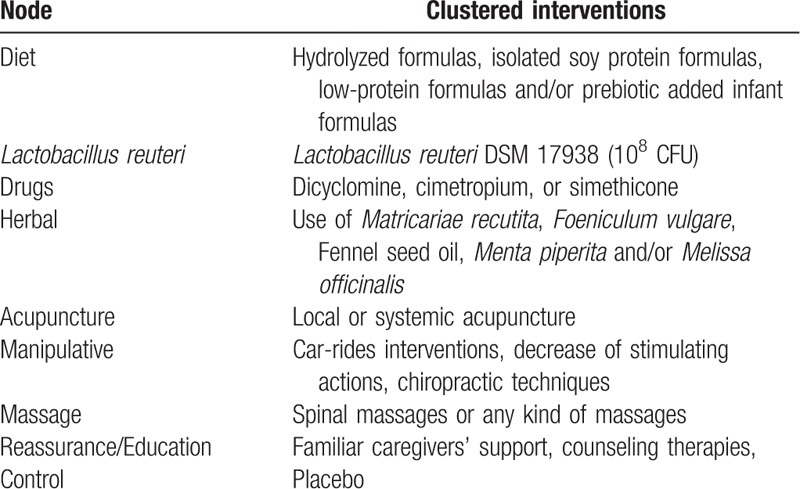
Intervention included in the node for analysis.

### 2.3 Data extraction and quality analysis

Quality evaluation of studies was performed in pairs, in a blinded and independent fashion using CONSORT statement for RCTs.^[58]^ Any discrepancy in the evaluation of the articles was resolved using Delphi methodology, which was coordinated by the principal investigator. Analyzed data included the research setting, the source of funding, characteristics of participants (age, gender, baseline pathologies, duration, and intensity of colic prior to study entry), type of therapeutics (dose, duration, frequency) and reported outcomes.

### 2.4 Data synthesis and analysis

From a statistic point of view, the information was analyzed with the strategy of multiple treatment meta-analysis. Considering that the common denominator of the majority of the studies was the use of placebo as comparator, we decided to use this intervention as the central axis for direct comparisons. Dichotomous outcomes were analyzed with the total number of randomly assigned participants as the denominator. For the secondary analysis of efficacy, measured as a binary outcome, the outcomes for missing information were generated, assuming that all participants with missing data did not respond to treatment. When reported, information on participants that abandoned the studies was included in the analysis. For each potentially eligible study, descriptive statistics of the population characteristics and their results were reported, describing the type of comparison as well as the most important clinical and methodological variables. For each pairwise comparison (direct or indirect), the Hedges standard mean difference (SMD) was calculated for continuous numeric variables, whereas the respective odds ratio (OR) was calculated for dichotomous outcomes. Both were calculated with their respective 95% confidence interval (CI_95%_). The first meta-analysis was a paired comparison of all published studies. We used a random effect model, considering that different studies estimated different treatment effects. Concomitantly, we calculated *I*^2^ for heterogeneity and its corresponding *P* value. Thereafter we assembled a NMA, using a random effect model with a Bayesian approach^[59,60]^ and summarized the results using effect sizes and CI_95%_. We used the adjusted model as described by Salanti et al.^[61]^ Additionally, we calculated the probability of superiority for each “anti-colic” intervention through a SUCRA analysis and presented the results in a ranked graph.^[62]^ To estimate the inconsistency (discordance between direct and indirect evidence with a CI_95%_ that did not include zero), we calculated the difference between the direct and indirect estimates, taking as reference only the constructed indicators that had included a placebo.^[63]^ Finally, we adjusted the model with and without assumptions of consistency and compared the 2 models in terms of fit and parsimony.^[64]^ In the case of a significant inconsistency we investigated the distribution of clinical and methodological variables that might have been a potential source of heterogeneity or inconsistency in each group of specific comparisons. All analysis and graphic depictions were performed on the version 13 of STATA for Mac.

## 3 Results

**Figure 1:**
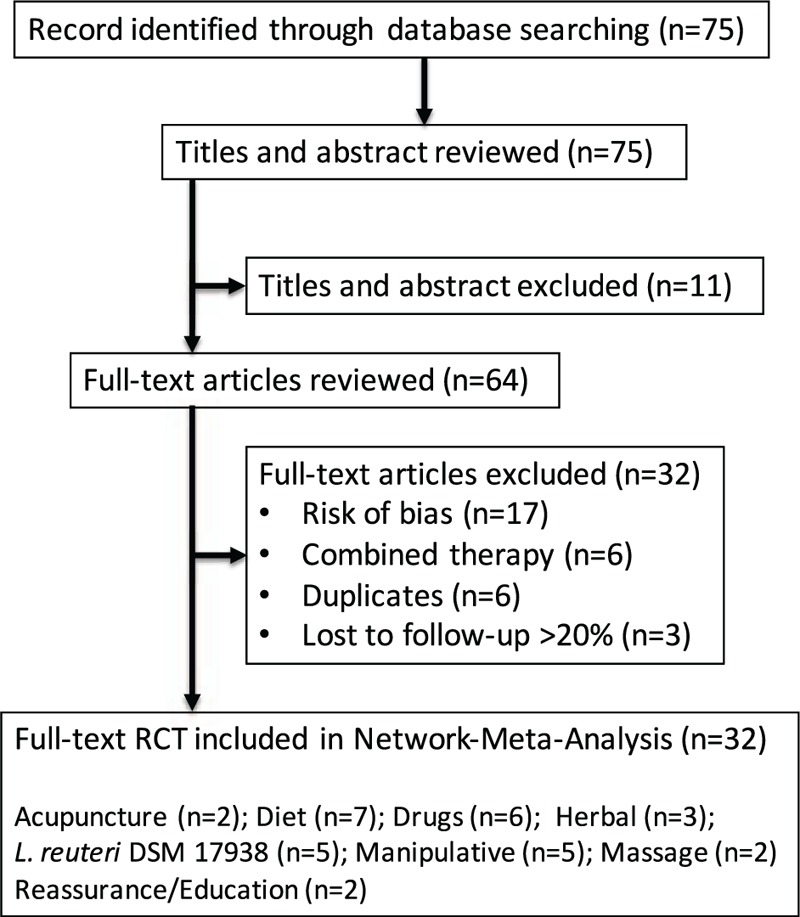
Flow chart of analyzed studies.

**Figure 2:**
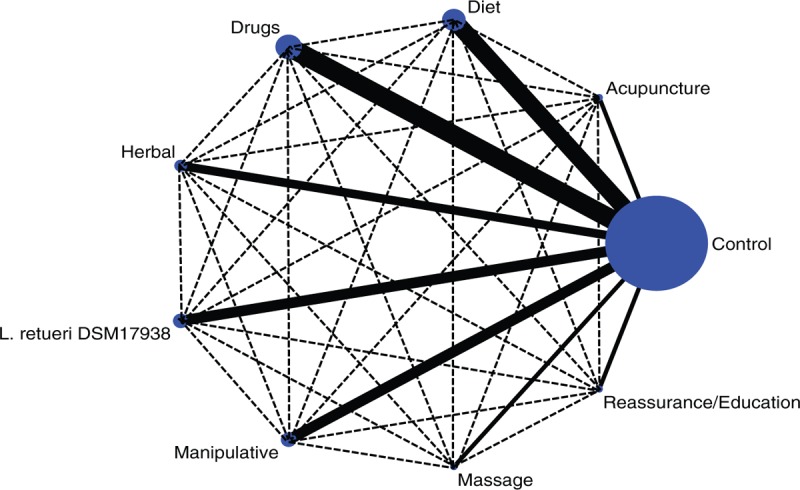
Network meta-analysis of multiple treatments for infantile colic.

**Figure 3:**
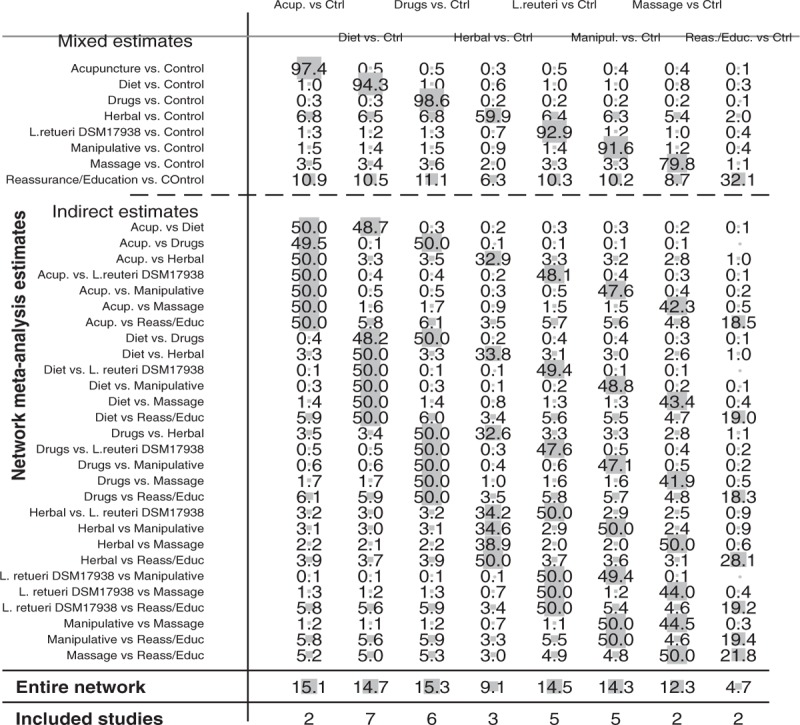
Contribution plot for the network meta-analysis.

**Figure 4:**
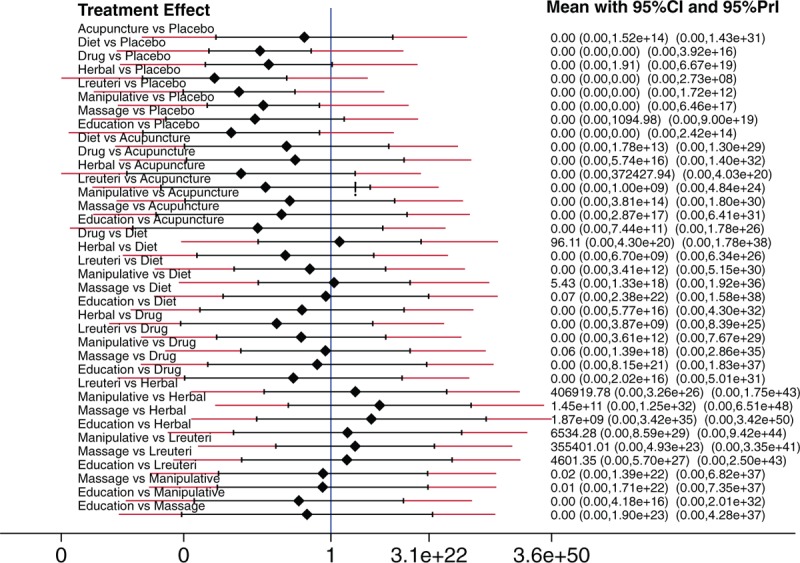
Forrest plot of multiple treatments for infantile colic.

**Figure 5:**
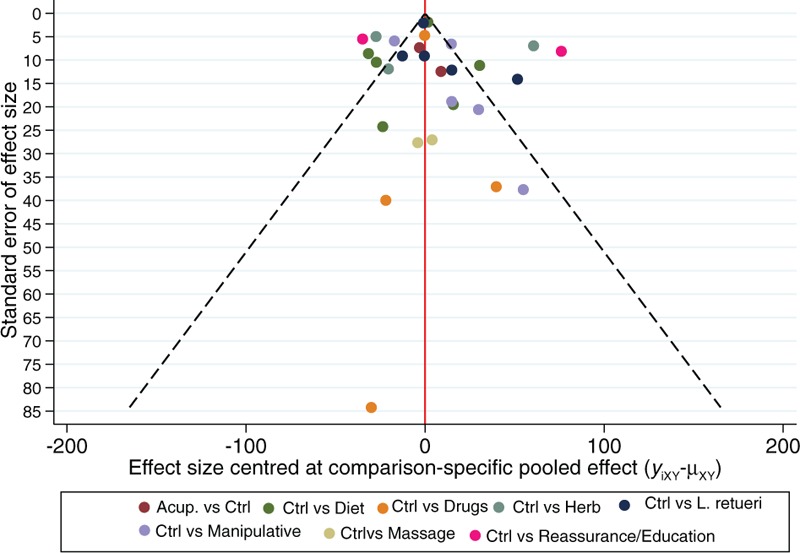
Comparison adjusted funnel plot of multiple treatments for infantile colic.

**Figure 6:**
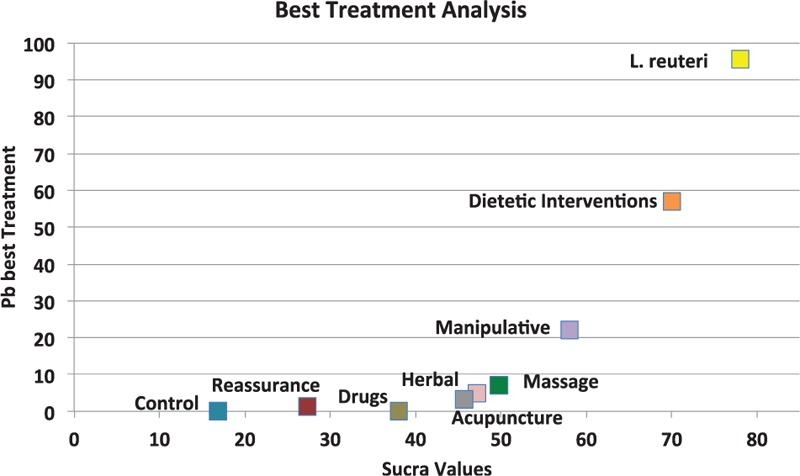
Ranking plot of multiple treatments for infantile colic.

About 32 RCTs were analyzed^[22–52]^ (Fig. 1), including 2242 patients randomized to 9 nodes of intervention (*L reuteri* DSM 17938, n = 175; dietetic and nutritional, n = 324; pharmacologic, n = 150; herbal, n = 133; acupuncture, n = 81; manipulative, n = 136; massage, n = 48; reassurance/education, n = 84 and placebo, n = 1,111) (Table 1 and Fig. 2). The RCTs were published between 1977 and 2015. sample sizes ranged from 10 to 111 patients per trial, with a median of 30. Fifty-six percent of total participants were females. The mean age of participants was 35 days (8 days–3 months). The number of visits during the study was 4 to 5 (Basal, day 7, 14, 21, and 28) (Table 2). The risk of bias was rated as low concerning randomized generation of the allocation sequence, allocation concealment and outcome evaluation for *L reuteri* DSM 17938 RCTs and moderate for the rest of RCTs. Through the contributive plot analysis, we were able to identify that studies with *L reuteri* DSM 17938 versus Ctrl., Diet versus Ctrl. and Acupuncture versus Ctrl. were the most influential studies in the NMA. The most informative direct evidence in the network was for these 3 comparisons, contributing with around 14% to 15% each one (Fig. 3). Regarding efficacy, considering the weighted mean differences (WMD) effect and the heterogeneity of studies we identify a superiority of *L reuteri* DSM 17938 [WMD −51.3 h (CI95% −72.2 to −30.5 h), *P* 0.0001] and dietetic approaches [WMD −37.4 h (CI95% −56.1 to −18.7 h), *P* 0.0001] (Table 3, Fig. 4). Through Forest plots of the network meta-analysis, we identify a low risk of bias (Fig. 5). Finally, we created hierarchies of effect size on the basis of SUCRA rankings for efficacy outcomes (Fig. 6). The best treatment, according to the curves, was *L reuteri* DSM 17938 and the least effective treatment was reassurance/education probably due to the high risk of bias identified on the 2 studies that we include in this analysis.

**Table 2:**
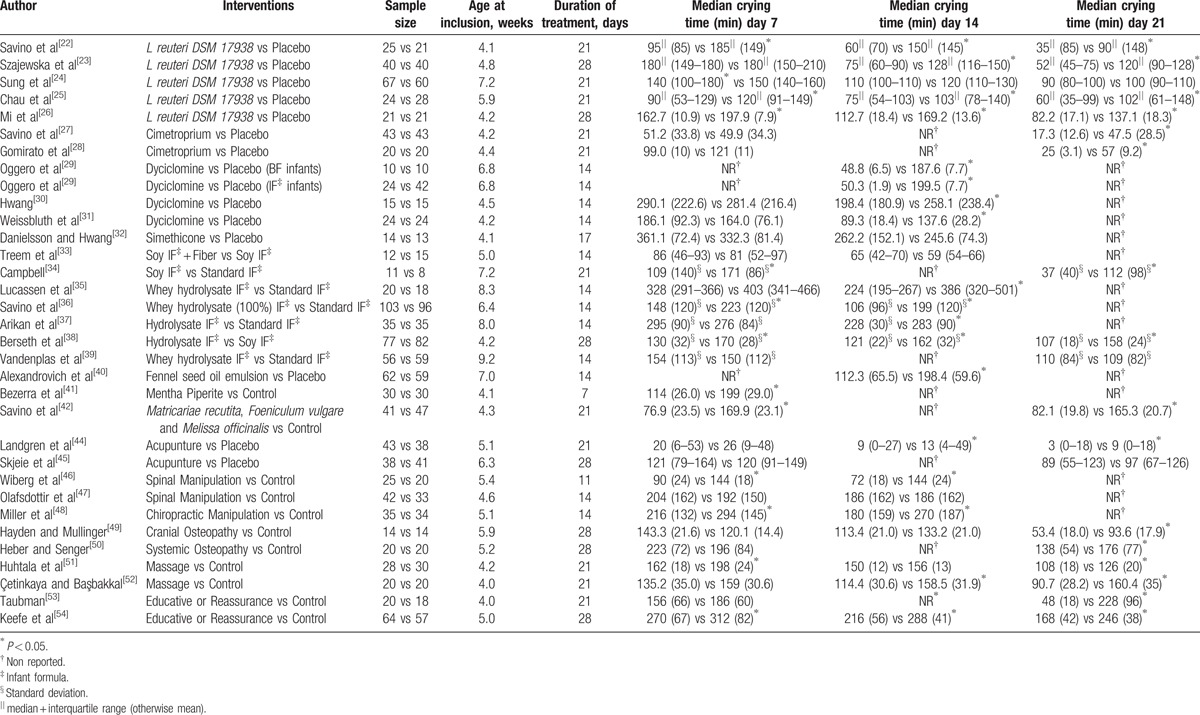
Characteristics of includes studies.

**Table 3:**
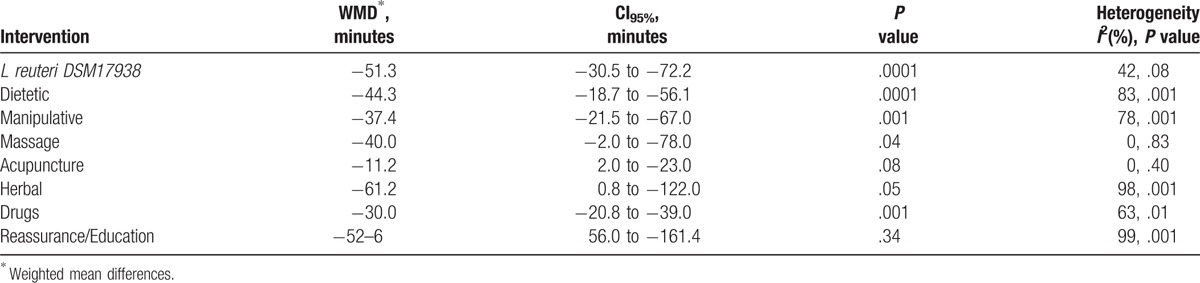
Comparative efficacy of treatments for infantile colic.

## 4 Discussion

Infantile colic is a common condition worldwide, affecting 1 in 5 infants younger than 3 months. Although infantile colic is considered to be self-limiting, it is often a stressful problem for parents and a frequent and wrongly undervalued digestive disorder.^[65]^ Recently, Indrio et al^[66]^ demonstrated that preventive intervention in infants not only reduce the probability of colic episodes, but also reduce the number of pediatric visits or visits to the emergency department due to digestive symptoms, the parent's absenteeism and the use of unproved intervention such as simethicone, cimetroprium or herbal products. Although a significant number of papers on infantile colic have been published for more than 45 years ago, there is no adequate consensus about the most efficient way to treat these patients and many times the interventions are selected based on experience more than evidence or analyzing the evidence with some bias. Evidence-based analysis using traditional approaches and single meta-analysis had demonstrated conflicting results when the different therapeutic options for colic had been evaluated.^[67]^ In this paper, we evaluated the evidence with the lowest risk of bias published regarding the treatment for infant with colic. We assembly a systematic review at first, searching the main databases around the world to reduce potential publication bias. After this approach and for the first time on this topic we used the NMA approach with the main purpose to establish direct and indirect comparisons, not only between active versus placebo, which is the most common analysis, but also establishing indirect comparison between active versus active treatments. From our point of view this is important because on the practical arena the clinical practitioners usually face the challenge to decide which treatment could be the best, but comparing one to other. We were able to demonstrate with this approach a superiority of the use of *L reuteri* DSM 17938 with a dose of 10^8^ CFU/day for 21 to 28 days to significantly reduce the duration of crying episodes during the day. This statement was supported with 4 homogeneous studies^[22,23,25,26]^ who consistently showed a reduction of the duration of colic in infants after the first 7 days of treatment. This superiority was demonstrated not only when we compared *L reuteri* DSM 17938 versus placebo, but also when we assembled the indirect comparisons with the other types of intervention, the superiority was maintained, as shown by the sucra analysis included in this paper. Additionally, we assembled a funnel plot analysis with the aim to demonstrate the absence of publication bias in this analysis. Multiple treatment analysis, assembled in this paper was important because, instead of different therapeutic options showed significant effects (i.e., dietetic, manipulative and herbal options), with *P*-values < 0.05, the NMA approach and the heterogeneity analysis demonstrated significant *I*^2^ values which reduced the possibility to recommend these types of treatments. Our findings about the superiority of *L reuteri* DSM 17938 for the treatment of infantile colic are strongly supported by some recently published hypothesis where different authors have identified different microbiota patterns in this type of children.^[10–16]^ Thereby new avenues are opened for continuing to establish new evidence in this field to support the use of a specific strain of probiotic, with a specific dose at a specific frequency as a potentially cost-effective treatment for these infants. Additionally, when you observed the sucra analysis it is important to identify the limited evidence that exists regarding some therapeutic options which are used frequently (simethicone, diclyclomine, herbal interventions) which must be cautions for practitioners before they decide to continue using these non-evidence-based interventions. Regarding limitations of this study we are clear that NMA assumes that treatment arms are similar in rationale and procedure, allowing us to group them together as one node in the network.^[61–64]^ However, we must be clear that decision to use for example reassurance or education could be slightly different when a decision to use probiotics or drugs are established. Additionally, instead of excluding studies with high risk of bias we identify some grades of heterogeneity and inconsistency among trials, which could have led to an overestimation of the effect size. Also, we did not establish a safety and/or cost-effectiveness approach which is also important at the moment of best make decisions.

## 5 Conclusions

*Based on systematic analysis of evidence and networking meta-analysis approach use of L reuteri* DSM 17938 seems to be the most evidence-based significant intervention to reduce the duration of crying time in infantile colic. [WMD −51.3 h (CI95% −72.2 to −30.5 h), *P* .0001]

Use of specialized infant formulas (i.e. partially hydrolyzed, whey-protein derivate) is the second most evidence-based intervention to reduce the clinical symptoms in this type of infants [WMD −37.4 h (CI95% −56.1 to −18.7 h), *P* 0.0001]

The associated evidence for the use of other interventions such as dicyclomine, cimetroprium, simethicone, herbals, acupuncture, or spinal massage is reduced or significantly biased to let us recommended as potential interventions.
