# Efficacy of Lactobacillus reuteri DSM 17938 for infantile colic: Systematic review with network meta-analysis: Erratum

**DOI:** 10.1097/MD.0000000000009730

**Published:** 2018-01-26

In the article, “Efficacy of Lactobacillus reuteri DSM 17938 for infantile colic: Systematic review with network meta-analysis”,^[1]^ which appeared in Volume 96, Issue 51 of *Medicine*, affiliation f should have appeared as “Biomolecules and Infant Health-EIMYT Lab, Instituto Nacional de Pediatría, Ministry of Health, Mexico.”

## References

[1] Gutiérrez-Castrellón P Indrio F Bolio-Galvis A Efficacy of Lactobacillus reuteri DSM 17938 for infantile colic: Systematic review with network meta-analysis. *Medicine*. 2017 96:e9375. 10.1097/MD.0000000000009375 PMC5758237 29390535

